# Temperature-Dependent Fecundity and Life Table of the Fennel Aphid *Hyadaphis foeniculi* (Passerini) (Hemiptera: Aphididae)

**DOI:** 10.1371/journal.pone.0122490

**Published:** 2015-04-30

**Authors:** Francisco S. Ramalho, José B. Malaquias, Aline C. S. Lira, Flávia Q. Oliveira, José C. Zanuncio, Francisco S. Fernandes

**Affiliations:** 1 Unidade de Controle Biológico, Embrapa Algodão, Campina Grande-Paraíba, Brazil; 2 Universidade Estadual Paulista——Jaboticabal, São Paulo, Brazil; 3 Universidade Federal de Viçosa, Viçosa, Minas Gerais, Brazil; CSIRO, AUSTRALIA

## Abstract

*Hyadaphis foeniculi* (Passerini) (Hemiptera: Aphididae) is a cosmopolitan species and the main pest of fennel in northeastern Brazil. Understanding the relationship between temperature variations and the population growth rates of *H*. *foeniculi* is essential to predict the population dynamics of this aphid in the fennel crop. The aim of this study was to measure the effect of constant temperature on the adult prereproductive period and the life table fertility parameters (infinitesimal increase ratio (*r_m_*), gross reproduction rate (*GRR*), net reproduction rate (*R_0_*), finite increase ratio (λ), generation time (*GT*), the time required for the population to double in the number of individuals (*DT*), and the reproduction value (*RV_x_*)) of the fennel pest *H*. *foeniculi*. The values of lx (survival of nymphs at age **x**) increased as the temperature rose from 15 to 28°C and fell at 30°C, whereas mx (number of nymphs produced by each nymph of age **x**) increased from 15 to 25°C and fell at 28 and 30°C. The net reproduction rates (*R_0_*) of populations of *H*. *foeniculi* increased with temperature and ranged from 1.9 at 15°C to 12.23 at 28°C for each generation. The highest population increase occurred with the apterous aphids at 28°C. The rate of population increase per unit time (*r_m_*) (day) ranged from 0.0033 (15°C) to 0.1995 (28°C). The highest values of *r_m_* were recorded at temperatures of 28°C and 30°C. The *r_m_* values were a good fit to the models tested, with R^2^ > 0.91 and R^2^_adj_ > 0.88. The models tested (Davidson, Sharpe and DeMichele modified by Schoolfield et al., Logan et al., Lamb, and Briere et al.) were very good fits for the *r_m_* values observed, with R^2^ > 0.91 and R^2^_adj_ > 0.88. The only exception was the Davidson model. Of the parameters studied, the reproductive capacity was higher in the apterous aphids, with the unique exception of daily fecundity at 28°C, which was higher in the alate aphids of *H*. *foeniculi*. Parameters relating to the age-specific fertility table for *H*. *foeniculi* were heavily influenced by temperature, with the highest biotic potential and population growth capacity found at 34°C. Therefore, the results obtained in this study could be of practical significance for predicting outbreaks of fennel aphids and improving the management of this aphid in fennel crops.

## Introduction

Intercropping cotton (*Gossypium hirsutum* Linné) with fennel (*Foeniculum vulgare* Miller) is a widely used strategy in northeastern Brazil for minimizing pest damage [1–3]. However, outbreaks of *Hyadaphis foeniculi* (Passerini) (Hemiptera: Aphididae) can significantly affect the yield and the quality of the fennel seeds [3]. *H*. *foeniculi* is a cosmopolitan species and the main pest of fennel in northeastern Brazil [4]. The outbreaks occur primarily during flowering, but can occur soon after the plants emerge [2,3]. The aphids suck the sap from the plant causing the flowers and fruits to wilt and dry up. The aphids indirectly cause mold to form as a result of honeydew excretion [4]. In the State of Paraiba, the highest incidence of this aphid usually occurs during the hottest periods (October to December) when it colonizes the inflorescences [2,3]. These aphids can reduce seed yields by up to 80% in monocultures [2]. Studies on the bioecology of this pest are crucial for optimizing control strategies.

To develop effective integrated control programs, it is essential that we understand the population dynamics of this pest [3,5]. We know that population dynamics of arthropods are temperature-dependent [6]. Furthermore, because temperature is considered the most important abiotic factor affecting biological processes and aphid development and reproduction rates [7,8], fertility life tables for insects subjected to a wide range of temperatures are appropriate tools for describing these dynamics [9,10]. Life tables have been described for various aphid species [11–15].

The temperature-dependent development of the fennel aphid means that limitations and thermal constants can be established for these insects, with time quantified in degree-days [16]. Therefore, we can predict the outbreak of an arthropod pest, such as aphids in agricultural systems [17,18]. This information will help us make decisions on how to implement integrated pest control programs [19], predict population peaks, and establish the timing for sampling operations and ecological zoning [20].

Understanding the relationship between temperature variations and the population growth rates of *H*. *foeniculi* is essential for predicting the behavior of this aphid in the fennel crop. According to Chattopadhyay et al. [21], to implement efficient, economic and ecological control of aphids, we must use climatic factors to determine the right time to control them and to allow producers to take opportune measures for efficient crop management.

The biological responses of *H*. *foeniculi* to different temperatures and its thermal requirements under laboratory conditions were reported by Malaquias et al. [22]. However, quantitative information on life table parameters, such as intrinsic rate of increase (*r*_*m*_), net reproductive rate (*R*_*0*_), finite rate of increase (*λ*), mean generation time (*GT*), and population doubling time (*DT*) were not published in their studies.

Life tables are power full tools for analyzing and understanding the impact of external factors such as temperature on the growth, survival, reproduction, and rate of increase of insect populations [13]. Thus in order to develop a better understanding of the variation in demography of this pest, it is necessary to develop accurate life tables for *H*. *foeniculi* under different temperatures. Understanding the demography of an insect under different temperatures is the cornerstone for developing strategy to manage it in an eco-friendly manner [13]. Therefore, the aim of this study was to measure the effect of constant temperature on the prereproductive period and the fertility life table parameters (infinitesimal increase ratio (*r*_*m*_), gross reproduction rate (*GRR*), net reproduction rate (*R*_*0*_), finite increase ratio (*λ*), generation time (*GT*), the time required for the population to double in the number of individuals (*DT*), and the reproduction value (*RV*_*x*_) of the fennel pest *H*. *foeniculi*. The information from this study will be useful in making strategic pest control decisions.

## Materials and Methods

### Insects and the fennel cultivar

The apterous and alate adults of *H*. *foeniculi* were used in our study. The apterous specimens were obtained from alate individuals collected from fennel (*F*. *vulgare*) crops of the Montadas cultivar at Embrapa Algodão, Campina Grande, Paraiba, Brazil (latitude 7° 11’ 56”, longitude 35° 52’ 32”, elevation 635 m, and temperature range 15–35°C). Alatoid 4th-instar nymphs (alate aphid precursors) were also collected in the fennel crops. Precursor individuals of the apterous and alate 4th-instar nymphs were characterized based on the absence or presence of wing pads. The aphids were kept in incubators at 25°C, a relative humidity of 70 ± 10% and a photophase of 12 h. They were placed in 100-ml plastic containers each containing an 8-cm leaf taken from a fennel plant in the vegetative state.

An end of each leaf was kept in a 2.5-ml plastic tube (usually used for dental anesthetic) filled with water to keep the leaf fairly turgid. The end of the plastic tube was sealed with water absorbent cotton wool to prevent leakage. The tube was inserted into the middle of the plastic container through a circular hole 3.1 cm in diameter. The water and leaves were replaced every day.

Field permissions were not required or obtained for sample collection. The field studies did not involve endangered or protected species.

### Thermal time bioassay

The insects were subjected to the constant temperatures of 3, 15, 20, 25, 28, 30 and 33°C, with a 12-h photoperiod and a relative humidity of 70 ± 10%. The choice of temperatures was based on the field data. Four blocks were used for each temperature, each block with 50 insects, for a total of 200 recently emerged nymphs (0–24 h) for each temperature. They were kept in 100 mL plastic receptacles with an 8 cm long leaf from *F*. *vulgare* plants (Montadas cultivar) in the vegetative state. A piece of black paper was placed inside each plastic receptacle to make the exuviae (remains of exoskeleton) easily visible.

The leaf stem was placed in a 2.5 ml plastic tube (usually used with dental anesthetics) filled with water and sealed with water-absorbent cotton wool to prevent leakage. The tube was inserted into the plastic receptacle through a hole in the center of the cap, 3.1 cm in diameter. The water and leaves were replaced daily. Observations were made at 12-h intervals using a stereoscopic microscope.

#### Reproductive parameters for H. foeniculi

The prereproductive period for *H*. *foeniculi* was determined as the interval between adult emergence and the production of the first offspring. The number of nymphs deposited by each female and the survival of nymphs and adults were recorded every 12 h.

### Fertility life table parameters for *H*. *foeniculi*

The parameters for the fertility life tables of *H*. *foeniculi* apterous wingless adults were calculated using the data obtained at each of the temperatures tested. The infinitesimal increase ratio (*r*_*m*_) (the rate of population increase per unit time) was calculated using the equation in Lotka [23]:

$$\sum_{x=0}^{y}\text{exp}(-rm.x)lx.mx=1$$

number of females produced by each female of age **x,** and **lx** = survival of females at age **x.** The gross reproduction rate (GRR) (the number of females produced by a single female during her lifetime, disregarding the survival of immature forms) was obtained using **mx** [24]. The net reproduction rate (*R*_*0*_) (the number of females produced by a single female during her lifetime) was calculated using the formula in Krebs [25]:

$$R_{0}=\sum_{x=0}^{y}lx.mx$$

The finite increase ratio (λ) (the number of females added to the population per female per unit time) was calculated using the formula in Krebs [25]:

$$\lambda =\text{antilog}(r_{m}x0.4343)$$

The generation time (*GT*) (the time elapsed between the birth of the parents and the birth of the offspring) was obtained using the formula in Krebs [25]:

$$GT=\text{ln}(R_{0})/r_{m}$$

The time necessary for the population to double (*DT*) was calculated using the formula in Krebs [25]:

$$DT=\text{ln}(2)/r_{m}$$

According to Krebs [25], the reproduction value (*RV*_*x*_) is the contribution that a female of age **x** will make to the future population. It was calculated for each age class (1 day), using the formula below:

$$RV_{x}=\sum_{t=x}^{y}\left(\frac{l_{t}}{l_{x}}\right)m_{t}$$

where **x** = base age class, **y** = oldest age class, **t** = any age class between **x** and **y**, **m**_**t**_ = number of females produced per female at time **t**, and l_t_/l_x_ = probability of living from age **x** to age **t**.

### Model assessment

When the r_*m*_ values were plotted for the temperatures tested, the distribution was found to be nonlinear. Therefore, we attempted to find an equation that better described the relationship between the infinitesimal increase ratio (*r*_*m*_) of *H*. *foeniculi* and the temperature. We applied five models (Davidson [26,27], Sharpe and DeMichele [28] modified by Schoolfield et al. [29], Logan et al. [6], Lamb [30] and Briere et al. [31]) that were used previously by Malaquias et al. [32] to describe the development of *H*. *foeniculi* as a function of temperature. Nonlinear model parameters were estimated using the Marquadt method [33], using PROC NLIN [34]. The models were assessed on the basis of the coefficients of determination (R^2^), the adjusted coefficients of determination (R^2^_ajust_), the residual sum of squares (RSS), and the corrected Akaike information criterion (AIC_c_) [35]. The highest values of R^2^ and R^2^_ajust_ and the lowest values of RSS and AIC_c_ indicated the best fit for the relationship between the *r*_*m*_ of *H*. *foeniculi* and the temperature. The corrected AIC was calculated using the formula: AIC_c_ = n log (RSS/n) + 2Kn/(n-K-1), where n = sample size, RSS = residual sum of squares, and K = number of parameters in the model, including an error term (number of free parameters in the model).

### Data analyses

To satisfy the prerequisites for the analysis of variance, the data on the prereproductive period, nymphs/female, nymphs/female/day and longevity for *H*. *foeniculi* were tested for normality and homoscedasticity. When necessary, the data were transformed with the square root of x. For comparing the reproductive variables among alate and apterous individuals and among temperatures, we used the Student-Newman-Keuls (SNK) test [36]. The data on the reproductive variables as a function of temperature were subjected to polynomial regression analysis. From the regression equations, we determined the maximum and minimum points on the curve. For calculating the life table parameters, we used PROC Lifetable [37]. The Jackknife method [38] was used for comparing the parameters among groups. The biological parameter means were calculated and compared among the treatment pairs using the Student t-test.

## Results

### Reproductive parameters for *H*. *foeniculi*

**Fig 1:**
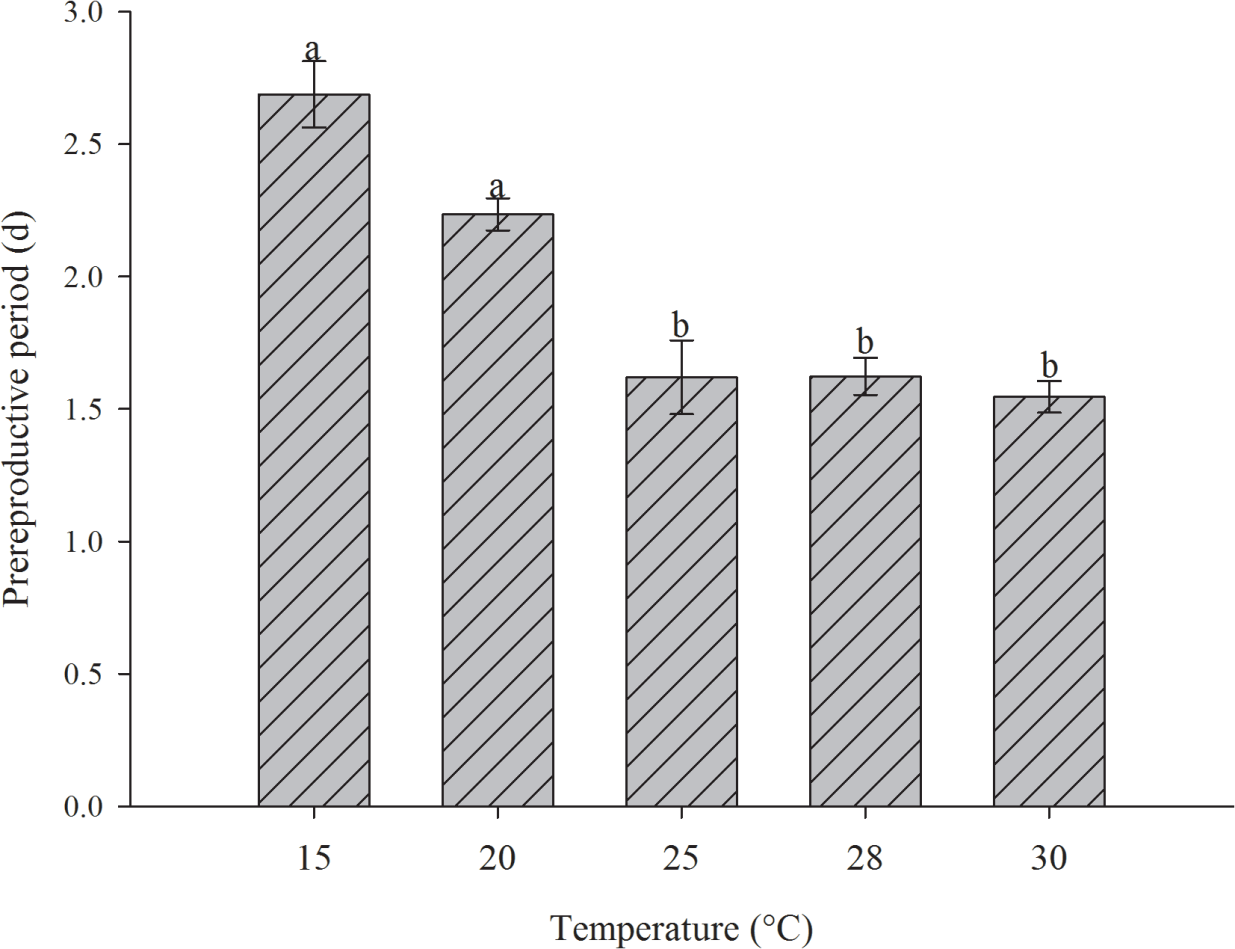
Prereproductive period (Mean ± SEM) for apterous morphs of *H*. *foeniculi* at different temperatures. Means with common lower case letters are not significantly different, the Student-Newman-Keuls test (*P* = 0.05). Standard error of the mean (SEM) is represented above each bar.

Temperatures between 15 and 30°C allowed the development of nymphs of all instars of *H*. *foeniculi*. Temperatures of 3 and 33°C were lethal to the aphid. Temperature had a significant impact on the prereproductive period of apterous individuals (*F* = 12.58, *df* = 4, 12, *P* = 0.0001), and the period ranged from 1.55 days at 30°C to 2.69 days at 15°C (Fig 1). The periods were shorter for the aphids at 25, 28 and 30°C than for the aphids at 15 and 20°C. However, there were no significant differences in the prereproductive periods of aphids kept at 15 and 20°C (Fig 1).

The temperature also significantly affected the reproductive parameters (mean number of nymphs produced per nymph, mean number of nymphs produced per nymph per day) and the longevity of both alate and apterous nymphs (Table 1). Comparing the fecundity of apterous females at the various temperatures, the number of nymphs/female was significantly lower at 15°C than at the other temperatures (Table 1). For alate aphids, the production of nymphs/female was higher at 25°C (14 nymphs/female). At 20 (7 females) and 28°C (5 females), the alate females produced approximately two or three times fewer females than at 25°C (14 females) (Table 1). There were no significant differences in the production of nymphs/female among the adult alate and apterous females at 20 and 25°C. However, at 28°C, the production of nymphs per apterous female (14 females) was approximately three times higher than that of the alate females (5 females) (Table 1).

**Table 1:** Influence of temperature (mean ± SEM) on biological variables (nymph/female, nymph/female/day and adult longevity) of *H*. *foeniculi* at different temperatures.

Temperature (°C)	Biological variables								
	Nymph (n)/female		*F*, df, *P*	Nymph (n)/female/day		*F*, *df*, *P*	Longevity (d)		*F*, *df*, *P*
	Apterous	Alate		Apterous	Alate		Apterous	Alate	
15	4.00 ± 1.13 B	-	-	0.51 ± 0.13 B	-	-	8.50 ± 1.38 B	-	-
20	8.50 ± 0.96 Aa	7.30 ± 0.97 Ba	0.49, 1, 6, 0.5093	0.55 ± 0.04 Bb	1.82 ± 0.43 ABa	10.06, 1, 6, 0.0193	14.33 ± 1.08 Aa	6.08 ± 0.56 Bb	29.98, 1, 6, 0.0016
25	13.03 ± 1.08 Aa	14.10 ± 2.12 Aa	0.05, 1, 6, 0.8243	0.97 ± 0.03 Aa	1.24 ± 0.09 Ba	4.86, 1, 6, 0.0696	14.08 ± 0.83 Aa	10.23 ± 0.91 Aa	5.43, 1, 6, 0.0586
28	13.50 ± 2.25 Aa	5.36 ± 1.00 Bb	9.96, 1, 6, 0.0197	1.20 ± 0.09 Ab	1.71 ± 0.34 Aa	29.16, 1, 6, 0.0017	12.29 ± 0.07 ABa	4.15 ± 0.94 Bb	30.03, 1, 6, 0.0015
30	8.98 ± 0.39 A	-	-	0.98 ± 0.02 A	-	-	9.05 ± 0.33 B	-	-
F, df, *P*	6.83, 4, 15, 0.0024	6.19, 2, 9, 0.0204	-	8.26, 4, 15, 0.0010	4.75, 2, 9, 0.0390	-	5.08, 4, 15, 0.0086	9.96, 2, 9, 0.0052	-

Means within a column followed by the same upper case letter are not significantly different based on the Student-Newman-Keuls test (*P* = 0.05). n = number.

The temperature affected the daily production of nymphs for both apterous (F = 8.26, df = 4, 15, *P* = 0.001) and alate females (*F* = 4.75, df = 2, 9, *P* = 0.0390). The apterous females produced fewer female nymphs per day at 15 and 20°C than at 25, 28 and 30°C. At 25°C, similar numbers of nymphs per day were produced by the apterous and alate females (Table 1). However, at 20 and 28°C, each alate female produced more nymphs than each apterous female (Table 1).

The longevities of the apterous (F = 5.08, df = 4, 15, *P* = 0.0086) and the alate (F = 9.96, df = 2, 9, *P* = 0.0052) adults were affected by temperature. The apterous females lived significantly longer at 20 and 25°C than at 15 and 30°C (Table 1). We observed no significant differences in the longevities of apterous aphids at 28°C compared with apterous aphids kept at the other temperatures. For alate aphids, higher longevity was observed for females at 25°C (10 days), but there were significant drops in alate female longevity at 20°C (6 days) and 28°C (4 days) (Table 1). At 20 and 28°C, the apterous females lived longer than the alate females (Table 1), but at 25°C the apterous and alate female longevities were similar (Table 1).

**Fig 2:**
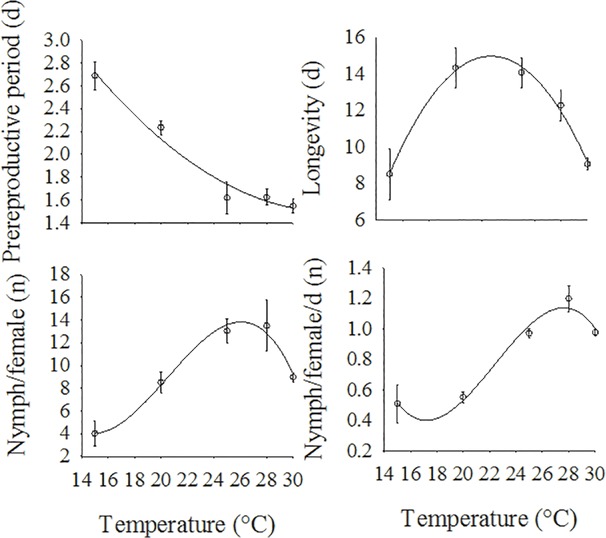
Effect of temperature on prereproductive period (y = 5.61–0.24x^2^ + 0.003x, F = 38.91, df = 2, 2, *P* = 0.0251, R^2^ = 0.9412), longevity (4.93x^2^ - 0.10x - 41.08, F = 97.69, df = 2, 2, *P* = 0.0101, R^2^ = 0.9816), nymph/female (-16.63x^3^ + 0.87x^2^ - 0.01x + 104.49, F = 157.33, df = 3, 1, *P* = 0.0467, R^2^ = 0.9409), and nymph/female/day (-1.86x^3^ + 0.08x^2^ - 0.001x + 13.10, F = 157.33, df = 3, 1, *P* = 0.0467, R^2^ = 0.9120) for apterous morphs of *H*. *foeniculi*. d = day.

Analyzing the regression curves for the reproductive parameters of apterous females as a function of temperature (Fig 2) we observed that the prereproductive period (*y* = 5.61–0.24x^2^ + 0.003x, F = 38.91, df = 2, 2, *P* = 0.0251, R^2^ = 0.9412) and longevity (*y* = 4.93x^2 -^ 0.10x - 41.08, F = 97.69, df = 2, 2, *P* = 0.0101, R^2^ = 0.9816) were better described by a quadratic function. However, the number of nymphs per apterous female (*y* = -16.63x^3^ + 0.87x^2^ - 0.01x + 104.49, F = 157.33, df = 3, 1, *P* = 0.0467, R^2^ = 0.9409) and the number of nymphs per apterous female per day (*y* = -1.86x^3^ + 0.08x^2^ - 0.001x + 13.10, F = 157.33, df = 3, 1, *P* = 0.0467, R^2^ = 0.9120) were better described by cubic functions. For all the parameters, we found good fits among the models because the estimates of *R* ^*2*^ were higher than 90%.

The longest prereproductive period (2.71 days) for apterous females was estimated using the model for a temperature of 15°C, whereas the shortest prereproductive period (1.52 days) was estimated for 30°C.

The model estimated the maximum fecundity of apterous females to be approximately 14 nymphs per apterous female at a temperature of 26.25°C (Fig 2). The highest daily production of apterous nymphs (1.13 nymphs/female/day) was estimated to occur at 27.59°C. For apterous female longevity, the highest point on the regression curve was at 22.73°C, with an estimated longevity of 14.98 days (Fig 2).

### Fecundity life table parameters for apterous *H*. *foeniculi*

**Fig 3:**
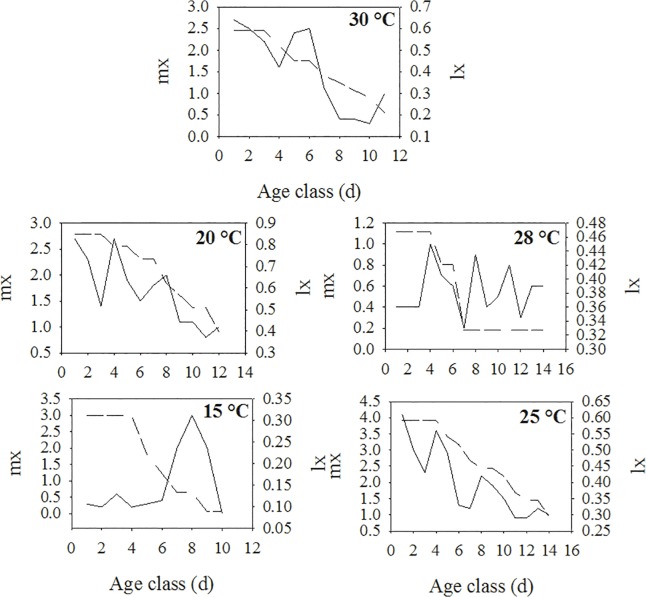
Relationship between age class (d) and age-specific daily fecundity (mx) and age-specific survival (lx) for apterous morphs of *H*. *foeniculi* at 15, 20, 25, 28 and 30°C. Age class = 1 d.

The values of lx and mx increased with temperature (Fig 3). The lx increased as the temperature rose from 15 to 28°C and then fell at 30°C, whereas the mx increased from 15 to 25°C and then fell at 28 and 30°C.

The net reproduction rates (*R*_*0*_) showed increases ranging from a factor of 1.9 at 15°C to 12.23 at 28°C for each generation in the populations of *H*. *foeniculi*. The highest population increase occurred for apterous aphids at 28°C. The rate of population increase per unit time (*r*_*m*_) ranged from 0.0033 (15°C) to 0.1995 (28°C). The highest values of *r*_*m*_ were recorded at temperatures of 28°C and 30°C (Table 2).

**Table 2:** Life table parameters for apterous morphs of *H*. *foeniculi* at five different temperatures.

Temperature (°C)	Life table parameters^1^				
	Net reproductive rate (*R*_*0*_)	Intrinsic rate of natural increase (*r*_*m*_)	Finite rate of increase *(λ)*	*GT* (d)	*DT* (d)
15	1.9037 c	0.0033 c	1.0340 c	19.2333 ab	20.7073 a
20	4.3440 b	0.0624 c	1.0644 c	23.5108 a	11.0950 a
25	7.5144 b	0.1399 b	1.1501 b	14.4148 b	4.9541 b
28	12.2328 a	0.1995 a	1.2208 a	12.5509 b	3.4741 b
30	5.2860 ab	0.1758 ab	1.1922 ab	9.4671 c	3.9410 b

^1^Means with the same lower case letter within a column do not differ significantly based on the *t*-test (*P* = 0.05). *GT (d) =* generation time in days and *DT* (d) = population doubling time in days.

The finite increase ratio (*λ*) was 1.2208 for females who spent their adult lives at 28°C and 1.1922 for females at 30°C, with no significant difference. Furthermore, the finite increase ratio (*λ*) observed at 30°C did not differ from the value of *λ* estimated for females kept at 25°C (Table 2). The lowest finite increase ratios were recorded at 15°C and 20°C (Table 2).

The mean generation time (*T*), the time elapsing between birth of the parents and birth of the offspring, ranged from 10 days (30°C) to 24 days (20°C) (Table 2).

The time required for the *H*. *foeniculi* populations to double (*TD*) ranged from 3 days (28°C) to 21 days (15°C). The estimated TD values for females kept at 15°C (21 days) and 20°C (11 days) were higher than those for females kept at 25°C (5 days), 28°C (3 days) or 30°C (4 days) (Table 2).

**Fig 4:**
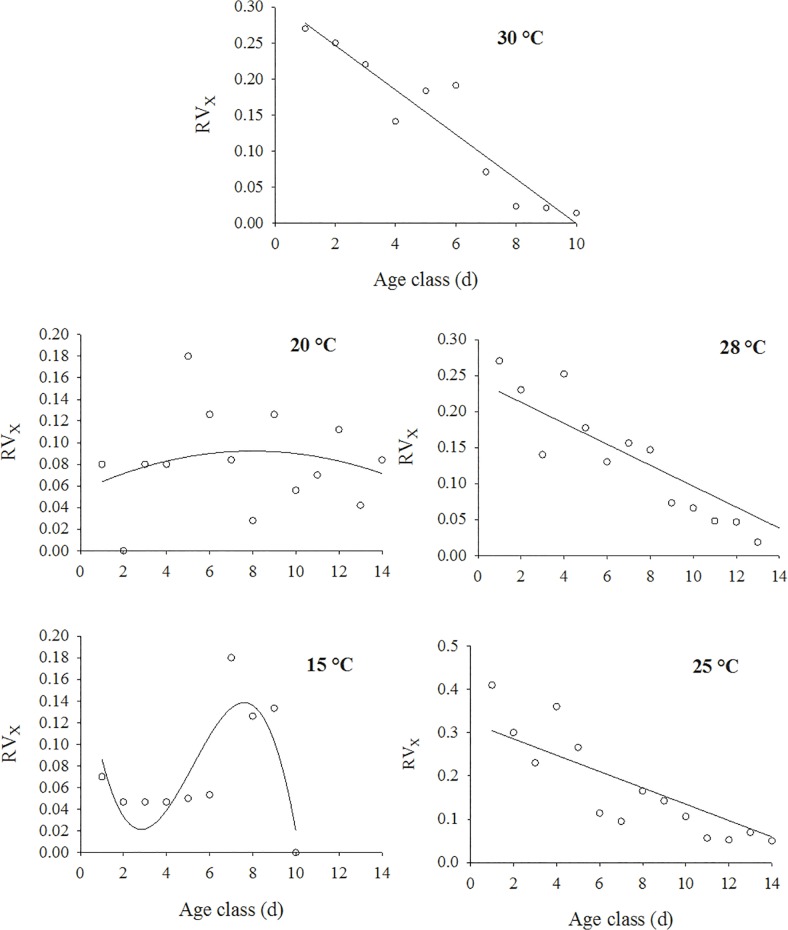
Reproductive value for apterous morphs of *H*. *foeniculi* as a function of temperature: 15°C (0.1939–0.1392x^3^ + 0.0337x^2^–0.0022x, *F* = 4.38, df = 3, 6, *P* = 0.0588, R^2^ = 0.6870); 20°C (0.0551 + 0.0093x^2^ - 0.0006x, F = 4.38, df = 3, 6, *P* = 0.0588, R^2^ = 0.6058); 25°C (0.3634–0.0254x, F = 43.29, df = 1, 12, *P* = 0.0001, R^2^ = 0.7829); 28°C (0.2712–0.0194x, F = 45.61, df = 1, 10, *P* = 0.0001, R^2^ = 0.8003); and 30°C (y = 0.3082–0.0308x, F = 64.93, df = 1, 8, *P* = 0.0001, R^2^ = 0.8903).

According to Krebs [25], the reproduction value (*RV*_*x*_) is the contribution that a female of age *x* makes to the future population. It was calculated for each age class and resulted in a cubic response at 15°C (Fig 4) and quadratic responses at the other temperatures (Fig 4). The lowest values of *R* ^*2*^ were observed at 15°C (R^2^ = 0.6870) and 20°C (R^2^ = 0.6058). At the other temperatures, the R^2^ values were higher than 80%. The highest values of *RV*_*x*_ were estimated for the first age class at the temperatures of 25, 28 and 30°C. However, at 20 and 15°C, the highest estimated values of *RV*_*x*_ were for the age classes 7 and 8, respectively (Fig 4).

### Model Assessment

**Fig 5:**
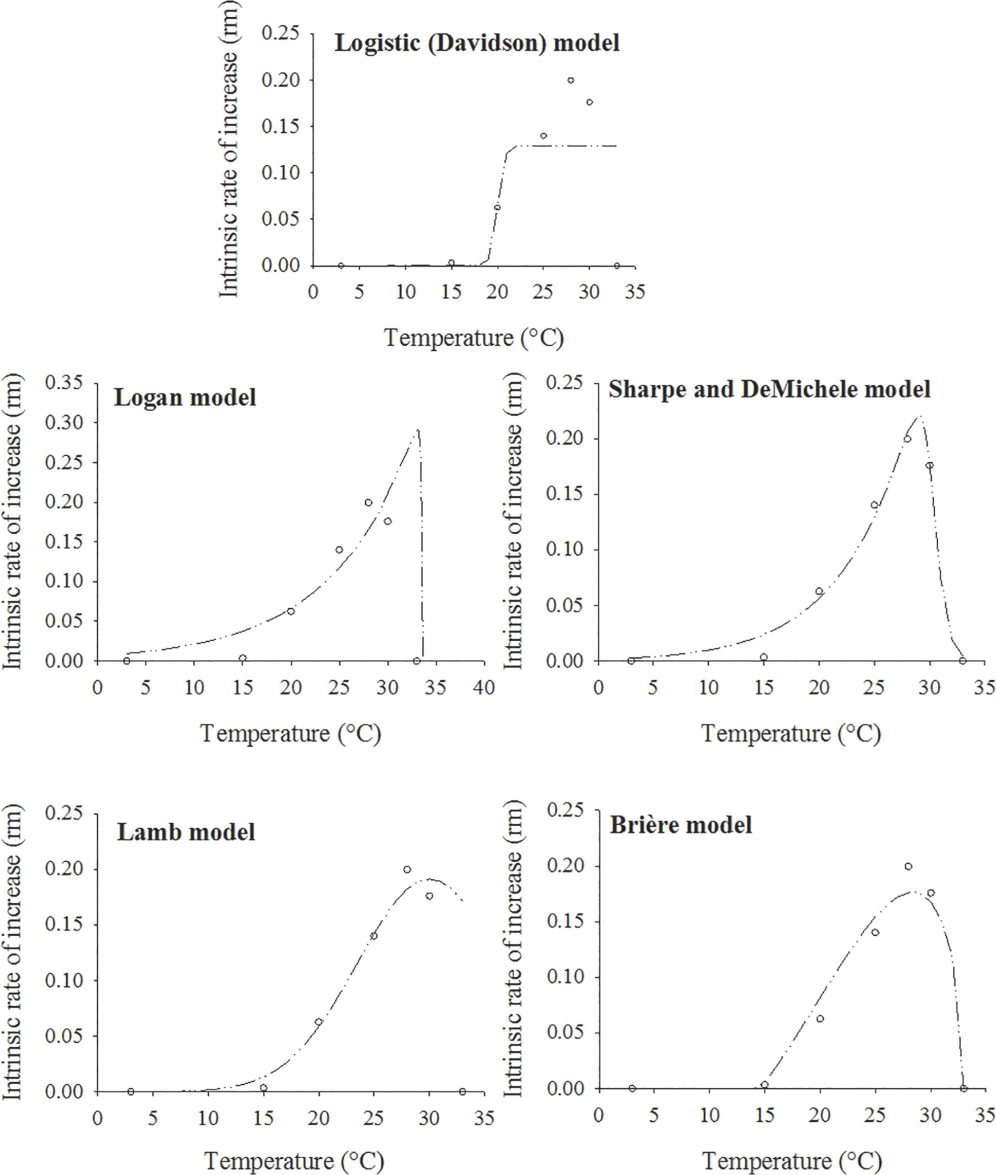
Fitting nonlinear models to the observed values of the increase rate (*r*_*m*_) for apterous morphs of *H*. *foeniculi* as function of temperature (°C). Observed (dots) and predicted data (dashed line).

The models tested were very good fits for the *r*_*m*_ values observed, with R^2^ > 0.91 and R^2^_adj_ > 0.88. The only exception was the Davidson model [26,27](Table 3 and Fig 5). The predicted optimum temperature ranged from 28.40°C (Brière-1 model [31]) to 33.08°C (Logan-6 model [6]) (Fig 5). The Sharpe and DeMichele [28] and Lamb [30] models provided the best fit to the data with higher R^2^ and R^2^_adj_ values and lower RSS values than the other models (Table 3). Nevertheless, based on the lowest values of the AIC_c_, the Sharpe and DeMichele [28] model (AIC_c_ = -66.59) was the best for describing the relationship between *r*_*m*_ and temperature. In general, the shapes of the curves estimated by the models used were almost identical, except for the Davidson model [26,27]. The Logan [6], Sharpe and DeMichele [28] and Lamb [30] models provided similar estimates of the lowest temperature (< 5°C). However, the curve of the Brière model [31] intersected the x-axis at approximately 15°C, indicating the lowest temperature. In the Logan [6], Lamb [30], Sharpe and DeMichele [28] and Brière [31] models, estimates of the highest temperature were similar, intersecting the x-axis at 33°C (Fig 5).

**Table 3:** Nonlinear models applied to describe the relationship between the intrinsic rate of increase (*r*_*m*_) for apterous morphs of *H*. *foeniculi* and temperature (°C).

Models	T_*rm*_ (°C)	RSS	F	*P*	R^2^	R^2^_adj_	AIC_c_
Logistic (Davidson)	23–33	0.023900	7.35	0.0325	0.4801	0.3826	- 32.95
Sharpe and DeMichele	29.00	0.000638	110.02	0.0014	0.9861	0.9780	- 66.59
Logan-6	32.90	0.003970	30.31	0.0033	0.9136	0.8827	- 59.75
Lamb	29.86	0.000652	107.61	0.0014	0.9858	0.9820	- 57.74
Brière-1	28.40	0.002800	43.56	0.0016	0.9391	0.9229	- 66.41

T_*rm*_ = predicted temperature at which the intrinsic rate of increase could reach its maximum, RSS = residual sum of squares, R^2^ = coefficient of determination, R^2^_adj_ = adjusted coefficient of determination, and AIC_c_ = corrected Akaike information criterion.

## Discussion

Life tables are important tools for analyzing and understanding the impact of external factors such as temperature on the growth, survival, reproduction and population growth of insects.

The temperature influenced the life history parameters of *H*. *foeniculi*. The temperatures of 3 and 33°C were lethal to apterous aphids. Alate aphids did not survive temperatures of 15 and 30°C. These results confirm the sensitivity of this aphid species to high and low constant temperatures in the laboratory [22,32]. At all other temperatures (15–30°C), all apterous adults were capable of reproducing. The alate aphids were capable of reproducing at 20–28°C.

The mean prereproductive period of *H*. *foeniculi* did not exceed three days. However, the shortest period was 1.55 days. Under temperature conditions ranging from 23 to 34°C, the prereproductive period for apterous individuals of *Hyadaphis tataricae* (Ajzenberg) (Hemiptera: Aphididae) was found to be only one day [38].

The prereproductive period of *H*. *foeniculi*, as a function of temperature, was best described using a quadratic polynomial model (Fig 2). According to De Conti et al. [39], the prereproductive periods of the aphids *Aulacorthum solani* (Kaltenbach), *Macrosiphum euphorbiae* (Thomas) and *Uroleucon ambrosiae* (Thomas) (Hemiptera: Aphididae), as functions of temperature, were also described using quadratic functions. There was a tendency for the prereproductive period of *H*. *foeniculi* to decrease as the temperature rose. However, for the aphids *A*. *solani*, *M*. *euphorbiae* and *U*. *ambrosiae*, the shortest prereproductive period was obtained at 16°C, with an estimated period of 3 days for *A*. *solani*, 2.8 days for *M*. *euphorbiae* and 1.8 days for *U*. *ambrosiae* [39].

The highest fecundity for alate females of *H*. *foeniculi* was found at 25°C (14 nymphs). At 28°C, nymph production for alate females was lower than that of apterous females (Table 1). The average fecundity of apterous females of *H*. *tartaricae* was 29.10 nymphs per female over a temperature range of 23–24°C [38] and was therefore higher than the figure we recorded in this study for *H*. *foeniculi*, and additionally, the fecundity of the alate *H*. *tartaricae* females was double that of the alate females at 25°C (14 nymphs). It is possible that allocating energy for the development and maintenance of wing muscles in alate aphids competes with the energy required for embryo development. This would account for the low fecundity of alate aphids that reproduce by viviparity, in contrast to apterous aphids as suggested by Dixon [40]. The fecundity observed for both apterous and alate female *H*. *foeniculi* was lower than that of other Aphididae reported in the literature. The maximum fecundity of apterous females of *H*. *foeniculi* was estimated with the regression model to be 14 nymphs, whereas for *Rhopalosiphum maidis* (Fitch) (Homoptera: Aphididae) and *Aphis glycines* Matsumura (Homoptera: Aphididae) fecundities exceeded 40 nymphs [41] and 60 nymphs [42], respectively. It is likely that *H*. *foeniculi* is better adapted to the temperature variations that occur in the field because these are the conditions under which frequent outbreaks of these aphids are observed in the fennel crops in northeastern Brazil [3]. Furthermore, the losses caused by *H*. *foeniculi* vary according to microclimatic conditions and the crop management system. For example, high losses were observed in monocultures with no ecological pest management [2]. According to Ramalho et al. [2], the resource concentration and natural enemies probably can account for reduced fennel aphid loads in fennel intercropped with cotton. Both resource concentration and natural enemies work together in regulating phytophagous insect populations [43].

The fecundity of *H*. *foeniculi*, as a function of temperature, was best described using a cubic model, with the maximum fecundity estimated at a temperature of 27.59°C (Fig 2). In various species of aphid, an increase in temperature often results in a drop in nymph production [44,45], as has been observed for the fecundity of *A*. *solani* and *U*. *ambrosiae*. There is evidence of a quadratic response to an increase in temperature for these species, with the high points on the fecundity curve estimated at 18.10°C (*A*. *solani)* and 17.40°C (*U*. *ambrosiae*) [39]. The different responses found for *A*. *solani*, *U*. *ambrosiae* and *H*. *foeniculi* are likely because *A*. *solani* and *U*. *ambosiae* are better adapted to low temperature conditions, whereas *H*. *foeniculi* is better adapted to higher temperatures, i.e., tropical regions. According to Sanchez et al. [46], during hot seasons, populations of *A*. *solani* and *U*. *ambosiae* fall drastically. In the fennel crops in northeastern Brazil, *H*. *foeniculi* were found with greater frequency during the hot season and were found between 153–188 days (apterous) and 139–174 days (alate) after transplanting in a monoculture and at 188 (apterous) and 195 days (alate) in an intercropped system [3].

The daily nymph production per apterous female of *H*. *foeniculi* was adversely affected by temperatures of 15 and 20°C. There was a difference in daily nymph production between apterous and alate females. It was higher in alate females at 20 and 28°C. In contrast, there was no difference between apterous and alate females of *H*. *tartaricae* [38]. The shorter longevity of alate *H*. *foeniculi* at 20 and 28°C may have contributed to the higher daily fecundity than that of apterous aphids. For alate aphids, the highest longevity was found in the females exposed to a temperature of 25°C. The peak on the longevity curve for apterous aphids was estimated at 22.73°C, with a lifetime of 14.98 days (Fig 2).

The biophysical factor of temperature significantly affected the biotic potential of *H*. *foeniculi*, as shown by the R_0_, r_*m*_, and *TD* parameters, as well as the population growth capacity as reflected by *r*_*m*_, the mean generation time and the time required for the population to double. For the net reproduction rate (*R*_*0*_), the highest capacity for increase was observed at 28°C and the lowest at 15°C. Aphids are known as “r-strategists”, i.e. they are very well adapted to exploiting a new temporary habitat by rapid population increase [47]. Their structure is simplified to enable them to perform best in feeding and reproduction, with most of their nutrients directed to reproduction [47]. However, under laboratory conditions, it is easy to underestimate the reproductive capacity because the highest value of *R*_*0*_ for *H*. *foeniculi* was only 12.23 (28°C). The variation of aphid responses to temperature could therefore be attributed to other factors, such as geographic origin and host plant [48,49]. Additionally, climatic changes can cause alterations in fennel seed concentrations of essential oils and terpenoid compounds [50], and thereby affect the feeding behavior of the pest species [51]. Furthermore, in the field, *H*. *foeniculi* can obtain larger quantities of nutritional complexes by continuously sucking sap or be more versatile and explore other resources available in addition to those contained in the leaves, such as the fructose and glucose present in the inflorescences [3]. In addition, the likely sensitivity to constant temperature (strain on *H*. *foeniculi* used in this study) could have contributed to lowering their reproductive capacity relative to that of the other Aphididae. The reproductive capacity (*R*_*0*_) of *Aphis gossypii* Glover (Hemiptera: Aphididae) was 57.07 at 25°C on *Cucumis sativus* Linné [49], whereas on *Capsicum annum* Linné, its *R*_*0*_ value was 59.43 at 30° [52]. An *R*_*0*_ value of 82.1 was recorded for individuals in a population of *A*. *gossypii* collected on *C*. *sativus* in Turkey [53].

The highest rates of population increase per unit time (*r*_*m*_) were found in apterous females at 28°C (r_m_ = 0.1995) and 30°C (r_m_ = 0.1758). In *A*. *gossypii*, this rate increased in direct proportion to temperature on two hosts, melon (*Cucumis melo* Linné) and cotton [54], but dropped significantly at temperatures above 30°C [51]. For the finite increase ratio (λ), the highest value for apterous *H*. *foeniculi* was found at 28°C, but was similar to that of aphids kept at 30°C. A direct proportional relationship between the finite increase ratio and the temperature (range 16–30°C) has been observed in *A*. *gossypii* [54].

For the mean generation times for *H*. *foeniculi*, the longest was 19.00 days (15°C) and the shortest was 9.00 days (30°C). For *A*. *gossypii*, this time ranged from 17.43 (15°C) to 5.63 days (30°C) [49]. The time required for the population of *H*. *foeniculi* to double at temperatures ranging from 25 to 30°C varied from 3.50 to approximately 5.00 days (28°C). Below 25°C, this time significantly increased. Similar results were obtained for *A*. *gossypii* [49,54].

The temperature affected the reproductive value of *H*. *foeniculi*. The reproductive value can be thought of as a key parameter in pest management programs because the pest’s reproductive behavior is a function of the specific age of the pest and indicates the best time to release the pest’s natural enemies [10]. In our study, the highest values of *RV*_*x*_ were estimated for the first age class at temperatures of 25, 28 and 30°C, whereas for the age classes 7 and 8, the highest values were estimated at 20 and 15°C, respectively. Other studies also found that temperature significantly affected the pattern of nymph production and the specific age of the insect. This was the case for *A*. *glycines* [44] and *Nasonovia ribisnigri* (Homoptera: Aphididae) [55].

Nonlinear models were also used by Nielsen et al. [56] to describe the relationship between *r*_*m*_ and temperature in *Halyomorpha halys* (Stal) (Hemiptera: Pentatomidae). In our study, the nonlinear models of Sharpe and DeMichele [28] and Lamb [30] provided the best fit for the relationship between *r*_*m*_ and temperature, with the highest values of R^2^ and R^2^_adj_ and the lowest values of RSS. However, according to Angilletta Jr [57], choosing models based on R^2^ and RSS may not necessarily be the best way to select a model. Nonetheless, based on the values of AIC_c_, the Sharpe and DeMichele [28] model was the best for describing the relationship between *r*_*m*_ and temperature for *H*. *foeniculi*. Malaquias et al. [32] found that the Sharpe and DeMichele model [28] was a good fit for estimating the development rates of *H*. *foeniculi* as a function of temperature. According to Wagner et al. [58], the Sharpe and DeMichele model [28] is one of the best for describing nonlinear responses involving insect development rates at extreme constant temperatures, as well as the linear relationships at intermediate temperatures. Other factors, such as intercropping fennel with cotton and the use natural enemies, have been reported to affect the populations of *H*. *foeniculi* [2]. According to Ramalho et al. [2], resource concentration and natural enemies most likely accounted for the reduced aphid loads on fennel intercropped with colored-fiber cotton. Both resource concentration and natural enemies work together to regulate plant feeding insect populations [43].

Climatic factors considerably influence the aphid pest populations [2]. Temperature is considered the most important abiotic factor affecting physiology [17], aphid reproduction rates [17] and, consequently aphid population dynamics [18]. However, we have little detailed understanding of stability and change in field populations of fennel aphids, but certain features of applied population dynamics have been revealed. Extraordinary weather conditions, particularly temperatures, can have a marked effect on fennel aphid numbers [22]. Natural enemies can influence the rate of buildup of fennel aphids [22] and by interacting with other factors can shape the dynamics of this fennel pest [3].

In conclusion, temperature significantly affected the prereproductive period in apterous aphids and the number of nymphs/female, the number of nymphs/female/day and the longevity of females in both alate and apterous individuals of *H*. *foeniculi*. Of the parameters studied, the reproductive capacity was higher in the apterous aphids, with the unique exception of daily fecundity at 28°C, which was higher in the alate aphids of *H*. *foeniculi*. The parameters relating to the age-specific fertility table for *H*. *foeniculi* were heavily influenced by temperature, with the highest biotic potential and population growth capacity found at 34°C. Therefore, the results obtained in this study maybe of practical significance for predicting outbreaks of fennel aphids and for improving the management of this pest in fennel crops. These data, combined with the monitoring of fennel aphid populations under field conditions, can also be effectively used to predict the parameters of *H*. *foeniculi* populations in fennel crops, and severe damage to the crop can be prevented by timely implementation of control measures. Models in this study require field testing before they can reach their full potential for predicting the population dynamics of *H*. *foeniculi*.

## Supporting Information

S1 Data Set Prereproductive period of *Hyadaphis foeniculi*. (DOC)

S2 Data Set Prereproductive period, longevity, nymph/female and nymph/female/day of *Hyadaphis foeniculi*. (DOC)

S3 Data Set *mx and lx at different temperatures* of *Hyadaphis foeniculi*. (DOC)

S4 Data Set Reproductive value (RVx) at different temperatures of *Hyadaphis foeniculi*. (DOC)

S5 Data Set Data for different models. (DOC)
