# Supplementary material for: Temperature-Dependent Fecundity and Life Table of the Fennel Aphid Hyadaphis foeniculi (Passerini) (Hemiptera: Aphididae)
Source: PLoS One. 2015 Apr 30;10(4):e0122490. doi: 10.1371/journal.pone.0122490 (PMC4415802; doi:10.1371/journal.pone.0122490)
Supplement: S1 Data Set — (DOC) [file pone.0122490.s001.doc]

**Data Set Fig. 1. Prereproductive period**

Temperature Mean SE

15.0000 2.6875 0.1250

20.0000 2.2348 0.0604

25.0000 1.6198 0.1393

28.0000 1.6229 0.0698

30.0000 1.5464 0.0598
