# Supplementary material for: Temperature-Dependent Fecundity and Life Table of the Fennel Aphid Hyadaphis foeniculi (Passerini) (Hemiptera: Aphididae)
Source: PLoS One. 2015 Apr 30;10(4):e0122490. doi: 10.1371/journal.pone.0122490 (PMC4415802; doi:10.1371/journal.pone.0122490)
Supplement: S2 Data Set — (DOC) [file pone.0122490.s002.doc]

**Data Set Fig. 2.** Prereproductive period, longevity, nymph/female and nymph/female/da*y* of *Hyadaphis foeniculi.*

*Prereproductive period*

**Temperature Mean SE**

15.0000 2.6875 0.1250

20.0000 2.2348 0.0604

25.0000 1.6198 0.1393

28.0000 1.6229 0.0698

30.0000 1.5464 0.0598

*Longevity*

**Temperature Mean SE**

15.0000 8.5000 1.3750

20.0000 14.3333 1.0833

25.0000 14.0825 0.8288

28.0000 12.2917 0.8646

30.0000 9.05000 0.3250

*Nymph/female*

**Temperature Mean SE**

15.0000 4.0000 1.1250

20.0000 8.5000 0.9583

25.0000 13.0325 1.0806

28.0000 13.5000 2.2500

30.0000 8.9750 0.3875

*Nymph/female/day*

**Temperature Mean SE**

15.0000 0.5107 0.1253

20.0000 0.5523 0.0354

25.0000 0.9700 0.0300

28.0000 1.1975 0.0863

30.0000 0.9765 0.0208
