# Supplementary material for: Temperature-Dependent Fecundity and Life Table of the Fennel Aphid Hyadaphis foeniculi (Passerini) (Hemiptera: Aphididae)
Source: PLoS One. 2015 Apr 30;10(4):e0122490. doi: 10.1371/journal.pone.0122490 (PMC4415802; doi:10.1371/journal.pone.0122490)
Supplement: S3 Data Set — (DOC) [file pone.0122490.s003.doc]

**Data Set Fig. 3.** mx and lx at different temperatures of *Hyadaphis foeniculi****.***

15°C

**Day mx lx**

1.0000 0.3000 0.3109

2.0000 0.2000 0.3109

3.0000 0.6000 0.3109

4.0000 0.2000 0.3109

5.0000 0.3000 0.2221

6.0000 0.4000 0.1777

7.0000 2.0000 0.1333

8.0000 3.0000 0.1333

9.0000 2.0000 0.0888

10.000 0.0000 0.0888

*20°C*

**Day mx lx**

1.0000 0.4000 0.4671

2.0000 0.4000 0.4671

3.0000 0.4000 0.4671

4.0000 1.0000 0.4671

5.0000 0.7000 0.4204

6.0000 0.6000 0.4204

7.0000 0.2000 0.3270

8.0000 0.9000 0.3270

9.0000 0.4000 0.3270

10.000 0.5000 0.3270

11.000 0.8000 0.3270

12.000 0.3000 0.3270

13.000 0.6000 0.3270

14.000 0.6000 0.3270

*25°C*

**Day mx lx**

1.0000 4.1000 0.5913

2.0000 3.0000 0.5913

3.0000 2.3000 0.5913

4.0000 3.6000 0.5913

5.0000 2.9000 0.5420

6.0000 1.3000 0.5174

7.0000 1.2000 0.4681

8.0000 2.2000 0.4435

9.0000 1.9000 0.4435

10.000 1.5000 0.4188

11.000 0.9000 0.3696

12.000 0.9000 0.3449

13.000 1.2000 0.3449

14.000 1.0000 0.2957

*28°C*

**Day mx lx**

1.0000 2.7000 0.8495

2.0000 2.3000 0.8495

3.0000 1.4000 0.8495

4.0000 2.7000 0.7929

5.0000 1.9000 0.7929

6.0000 1.5000 0.7362

7.0000 1.8000 0.7362

8.0000 2.0000 0.6230

9.0000 1.1000 0.5663

10.0000 1.1000 0.5097

11.0000 0.8000 0.5097

12.0000 1.0000 0.3964

*30°C*

**Day mx lx**

1.0000 2.7000 0.5912

2.0000 2.5000 0.5912

3.0000 2.2000 0.5912

4.0000 1.6000 0.5216

5.0000 2.4000 0.4521

6.0000 2.5000 0.4521

7.0000 1.1000 0.3825

8.0000 0.4000 0.3478

9.0000 0.4000 0.3130

10.000 0.3000 0.2782

11.0000 1.0000 0.2087
