# Supplementary material for: Temperature-Dependent Fecundity and Life Table of the Fennel Aphid Hyadaphis foeniculi (Passerini) (Hemiptera: Aphididae)
Source: PLoS One. 2015 Apr 30;10(4):e0122490. doi: 10.1371/journal.pone.0122490 (PMC4415802; doi:10.1371/journal.pone.0122490)
Supplement: S4 Data Set — (DOC) [file pone.0122490.s004.doc]

**Data Set Fig. 4.** *Reproductive value (RVx ) at different temperatures of Hyadaphis foeniculi****.***

*15°C*

**Day RVx**

1.0000 0.0700

2.0000 0.0467

3.0000 0.0467

4.0000 0.0467

5.0000 0.0500

6.0000 0.0533

7.0000 0.1800

8.0000 0.1260

9.0000 0.1333

10.000 0.0000

*20°C*

**Day RVx**

1.0000 0.0800

2.0000 0.0000

3.0000 0.0800

4.0000 0.0800

5.0000 0.1800

6.0000 0.1260

7.0000 0.0840

8.0000 0.0280

9.0000 0.1260

10.000 0.0560

11.000 0.0700

12.000 0.1120

13.000 0.0420

14.000 0.0840

15.000 0.0720

16.000 0.0120

17.000 0.0840

18.000 0.0600

19.000 4.0000e-3

20.000 2.0000e-3

21.000 0.0000

*25°C*

**Day RVx**

1.0000 0.4100

2.0000 0.3000

3.0000 0.2300

4.0000 0.3600

5.0000 0.2658

6.0000 0.1138

7.0000 0.0950

8.0000 0.1650

9.0000 0.1425

10.000 0.1063

11.000 0.0563

12.000 0.0525

13.000 0.0700

14.000 0.0500

15.000 0.0275

16.000 0.0375

17.000 0.0167

18.000 6.2500e-3

19.000 1.2500e-3

20.000 0.0000

*28°C*

**Day RVx**

1.0000 0.2700

2.0000 0.2300

3.0000 0.1400

4.0000 0.2520

5.0000 0.1773

6.0000 0.1300

7.0000 0.1560

8.0000 0.1467

9.0000 0.0733

10.000 0.0660

11.000 0.0480

12.000 0.0467

13.000 0.0187

14.000 8.0000e-3

15.000 8.0000e-3

16.000 0.0133

17.000 0.0400

18.000 0.0133

19.000 0.0000

*30°C*

**Day RVx**

1.0000 0.2700

2.0000 0.2500

3.0000 0.2200

4.0000 0.1412

5.0000 0.1835

6.0000 0.1912

7.0000 0.0712

8.0000 0.0235

9.0000 0.0212

10.000 0.0141
