# Supplementary material for: Temperature-Dependent Fecundity and Life Table of the Fennel Aphid Hyadaphis foeniculi (Passerini) (Hemiptera: Aphididae)
Source: PLoS One. 2015 Apr 30;10(4):e0122490. doi: 10.1371/journal.pone.0122490 (PMC4415802; doi:10.1371/journal.pone.0122490)
Supplement: S5 Data Set — (DOC) [file pone.0122490.s005.doc]

**Data Set Fig. 5.** Different Models.

*Temperature observed rm*

3.0000 0.0000

15.000 3.3000e-3

20.000 0.0624

25.000 0.1399

28.000 0.1995

30.000 0.1758

33.000 0.0000

*Logistic Davidson Model*

**Temperature predicted rm**

3.0000 1.0485e-22

4.0000 1.8177e-21

5.0000 3.1513e-20

6.0000 5.4631e-19

7.0000 9.4710e-18

8.0000 1.6419e-16

9.0000 2.8465e-15

10.000 4.9347e-14

11.000 8.5550e-13

12.000 1.4831e-11

13.000 2.5712e-10

14.000 4.4574e-9

15.000 7.7275e-8

16.000 1.3396e-6

17.000 2.3221e-5

18.000 4.0137e-4

19.000 6.6213e-3

20.000 0.0624

21.000 0.1213

22.000 0.1283

23.000 0.1288

24.000 0.1288

25.000 0.1288

26.000 0.1288

27.000 0.1288

28.000 0.1288

29.000 0.1288

30.000 0.1288

31.000 0.1288

32.000 0.1288

33.000 0.1288

*Logan Model*

**Temperature predicted rm**

3.0000 9.5587e-3

4.0000 0.0107

5.0000 0.0120

6.0000 0.0135

7.0000 0.0151

8.0000 0.0169

9.0000 0.0189

10.000 0.0212

11.000 0.0238

12.000 0.0267

13.000 0.0299

14.000 0.0335

15.000 0.0375

16.000 0.0421

17.000 0.0472

18.000 0.0528

19.000 0.0592

20.000 0.0664

21.000 0.0744

22.000 0.0834

23.000 0.0935

24.000 0.1047

25.000 0.1174

26.000 0.1316

27.000 0.1474

28.000 0.1652

29.000 0.1852

33.000 0.2902

33.621 0.0000

*Sharpe and DeMichele Model*

**Temperature predicted rm**

3.0000 2.6729e-3

4.0000 3.2307e-3

5.0000 3.8996e-3

6.0000 4.7008e-3

7.0000 5.6591e-3

8.0000 6.8038e-3

9.0000 8.1695e-3

10.000 9.7968e-3

11.000 0.0117

12.000 0.0140

13.000 0.0168

14.000 0.0200

15.000 0.0238

16.000 0.0284

17.000 0.0337

18.000 0.0401

19.000 0.0475

20.000 0.0563

21.000 0.0666

22.000 0.0788

23.000 0.0930

24.000 0.1097

25.000 0.1293

26.000 0.1521

27.000 0.1782

28.000 0.2059

29.000 0.2213

30.000 0.1762

31.000 0.0743

32.000 0.0193

33.000 4.3137e-3

*Lamb Model*

**Temperature predicted rm**

3.0000 3.3268e-5

4.0000 6.2421e-5

5.0000 1.1437e-4

6.0000 2.0464e-4

7.0000 3.5757e-4

8.0000 6.1012e-4

9.0000 1.0166e-3

10.000 1.6542e-3

11.000 2.6284e-3

12.000 4.0784e-3

13.000 6.1798e-3

14.000 9.1442e-3

15.000 0.0132

16.000 0.0186

17.000 0.0257

18.000 0.0346

19.000 0.0454

20.000 0.0583

21.000 0.0730

22.000 0.0894

23.000 0.1068

24.000 0.1246

25.000 0.1420

26.000 0.1580

27.000 0.1717

28.000 0.1822

29.000 0.1888

30.000 0.1911

31.000 0.1888

32.000 0.1822

33.000 0.1717

*Brière Model*

**Temperature predicted rm**

3.0000 0.0000

4.0000 0.0000

5.0000 0.0000

6.0000 0.0000

7.0000 0.0000

8.0000 0.0000

9.0000 0.0000

10.000 0.0000

11.000 0.0000

12.000 0.0000

13.000 0.0000

14.000 0.0000

15.000 5.1399e-3

16.000 0.0192

17.000 0.0341

18.000 0.0496

19.000 0.0655

20.000 0.0815

21.000 0.0975

22.000 0.1132

23.000 0.1281

24.000 0.1419

25.000 0.1542

26.000 0.1645

27.000 0.1720

28.000 0.1760

29.000 0.1752

30.000 0.1679

31.000 0.1508

32.000 0.1168

33.000 0.0000
